# The role of applying radiological modifiers to the Letournel classification and its clinical implications

**DOI:** 10.1038/s41598-026-42515-x

**Published:** 2026-04-04

**Authors:** Mohammad Kamal Abdelnasser, Mostafa A. Thabet, Bahaaeldin Ibrahim, Ahmed Abdelazim Hassan, Osama Farouk

**Affiliations:** 1 Orthopedic Department, Faculty of Medicine, Assiut University Hospital, Assiut University, Assiut, Egypt; 2 Orthopedic Department, Faculty of Medicine, Al-Azhar University, Assiut, Egypt

**Keywords:** Acetabular fractures, Classification, Letournel classification, Medical research, Trauma

## Abstract

While the Letournel classification is the most widely used system for classifying acetabular fractures, it has some limitations, such as a limited inclusivity and a limited ability to guide surgical approaches. Introducing radiological modifiers to the Letournel classification could address those shortcomings. The main aim of this study is to identify these modifiers, determine their incidence relative to different acetabular fracture patterns, and explore their association with fractures that cannot be classified using the Letournel system. The secondary objective is to evaluate how these modifiers may improve their utility in guiding surgical approaches. The radiographs and CT scans of 236 acetabular fractures were retrospectively reviewed by 2 authors to classify the fracture and to detect the presence of the radiological modifiers, which are roof impaction, marginal impaction, head impaction, head fracture, intraarticular fragments, preoperative dislocation and its type, articular comminution, pelvic ring involvement, and quadrilateral plate involvement. Using the modifiers, the number of unclassified fractures was reduced by 90%. Some modifiers were more significantly common in the older age group. The presence of specific modifiers, such as roof impaction and intraarticular fragments, mandated the use of a different approach. We used surgical hip dislocation in 21(8.8%) cases based on specific modifiers, namely, femoral head fracture 11, roof impaction 6, and intraarticular fragment 2, pure impaction (unclassified) 1, and labral avulsion with posterior rim in 1. Infrapectineal plating was done in 14 cases (6 %) based on the presence of quadrilateral plate modifier. Identifying the characteristics of an acetabular fracture is essential for enhancing the value of its classification. Given the complex anatomy and varied injury patterns of the acetabulum, an accurate description that includes radiological modifiers—such as posterior wall involvement or quadrilateral plate displacement—provides a more comprehensive assessment. Integrating these modifiers into the Letournel classification improves its ability to predict prognosis and guides surgical planning more effectively.

## Introduction

Accurate classification of acetabular fractures is essential for preoperative planning and choice of the surgical approach. Many classification systems have been proposed for acetabular fractures^1–5^; however, the Letournel classification, first published in 1964^1^ and updated in 1980^2^, is the most widely accepted. Yet, it has some drawbacks. Difficulty in mastering this classification system is a significant downside^6–9^. Inclusivity is another issue. Several case reports described unusual or unclassified patterns of acetabular fractures^10–17^. Similarly, many epidemiological studies and case series reported that unclassified fractures accounted for 1% to 35% of all acetabular fractures admitted to their institutions^18–20^.

Moreover, Letournel’s classification does not address some factors that may affect the outcome of acetabular fracture^21^ or guide the surgical approach. Those factors are called modifiers and are partly included in the comprehensive classification proposed by the AO group^4^. Yet, some other factors that may affect the choice of treatment method and surgical approach are not listed in the AO classification.

Surgical decision requires an accurate description of the fracture configuration. Many factors may affect decision-making in acetabular fracture management^22–24^, including the presence of roof impaction, intra-articular fragments, or quadrilateral plate involvement, which may alter the surgical approach. There is a need for a detailed classification system that can provide an accurate description of all fracture components, guide surgical decisions, and help to predict prognosis^25^.

The primary outcome of this study is to report the incidence of these modifiers in each acetabular fracture type, their relation to age, and whether there are relationships between different modifiers and fracture types. The secondary outcomes are to study the possible effects of these modifiers to reduce the number of unclassifiable fracture patterns and to guide the surgical approach.

## Methods

Between 1st January 2016 and 31st December 2017, 235 patients with 236 acetabular fractures were included in this study. Due to the retrospective nature of this study, the Assiut University Faculty of Medicine Institutional Review Board waived the need for informed consent and the experimental protocol. All procedures were performed in accordance with the relevant guidelines and regulations.

**Fig. 1:**
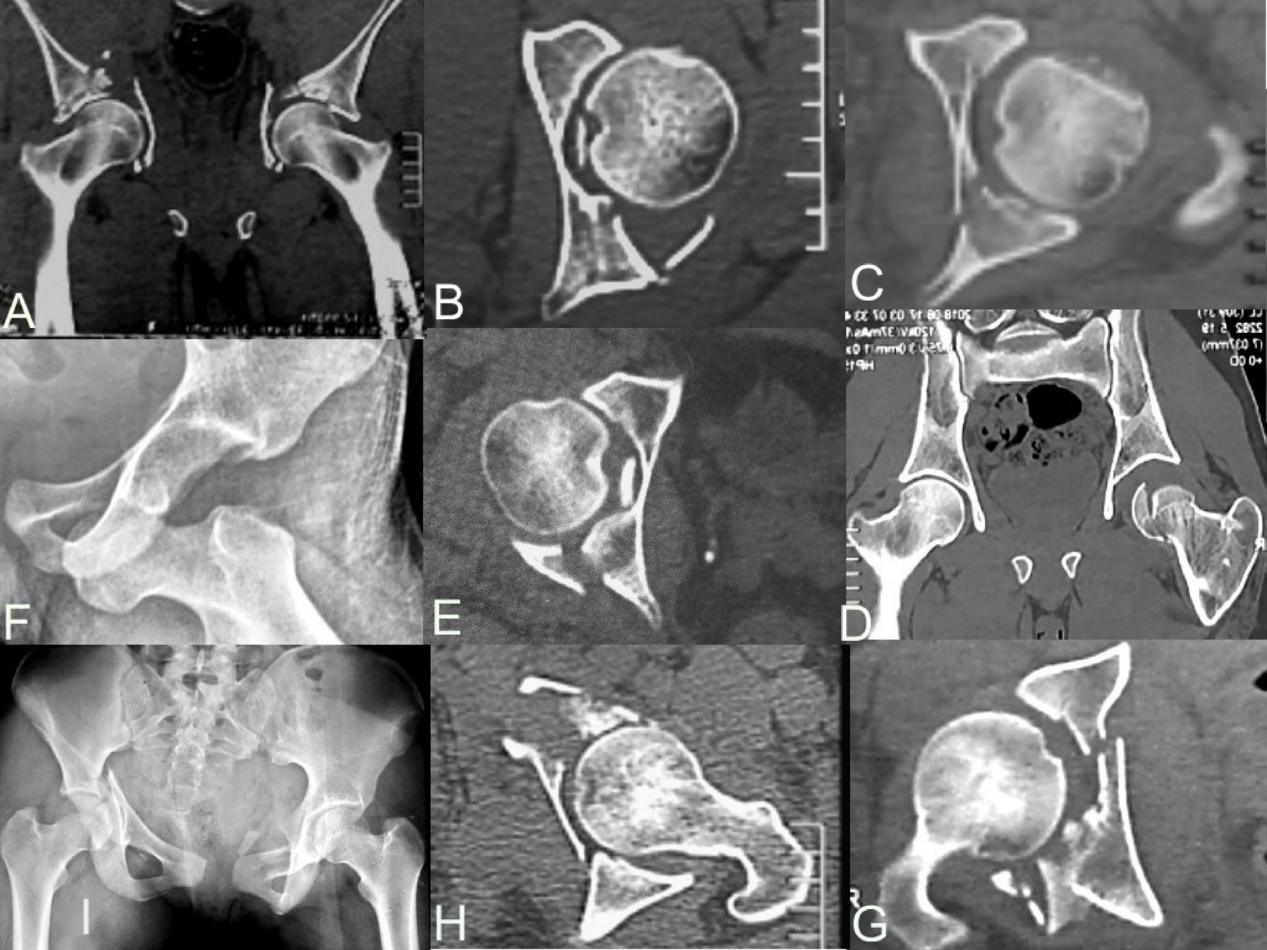
CT scans and plane radiographs showing various radiological modifiers: (**A**) roof impaction, (**B**) Marginal impaction, (**C**) femoral head impaction, (**D**) head fracture, (**E**) intra-articular fragment, (**F**) preoperative dislocation, (**G**) fracture site comminution, (**H**) Quadrilateral plate involvement, (**I**) pelvic ring involvement.

We set a number of radiological modifiers to be searched for in each fracture radiograph and CT scan by two experienced surgeons with 10- and 5-year experience in acetabular fractures, respectively. These modifiers are illustrated in Fig. 1.

1. Roof impaction is defined as subchondral impaction in the acetabular dome (weight-bearing area) as best viewed in the mid-coronal CT cut.
2. Marginal impaction is best visualised on a mid-axial CT cut.
3. Femoral head impaction (can be viewed in both radiographs and CT).
4. Femoral head fracture (can be viewed in both radiographs and CT).
5. Intraarticular fragments are best viewed in the mid-axial CT cut.
6. Preoperative dislocation and its type (Anterior, posterior, and central) are best viewed in both radiographs and CT.
7. Fracture site comminution (defined as more than three or more separate articular fragments as determined by CT scan).
8. Pelvic ring involvement (can be viewed in both radiographs and CT).
9. Quadrilateral plate (QLP) involvement is defined as fractures where the QLP is wholly or partially separated from the anterior and posterior columns of the acetabulum. QLP involvement was classified according to El Nahal`s classification, which classified QLP fracture into three types: incompletely separated simple quadrilateral plate fractures (QLP1), incompletely separated comminuted quadrilateral plate fractures (QLP2) and completely separated comminuted fractures (QLP3)^26^.
10. Associated posterior wall fracture with other types of acetabular fractures not listed in the Letournel classification system.

We studied the incidence of each modifier in the cohort. Also, the incidence of each modifier in each age group, and across different types of Letournel classification. We recorded how many times the surgical approach was modified based on a specific modifier and the reason for each modification.

### Statistical analysis

Data were analysed using PSS Statistics 21.0 (IBM SPSS Inc., Chicago, IL, USA). Descriptive statistics: Means, standard deviations, and percentages were calculated. Weighted Cohen’s Kappa agreement was used to test the inter-rater reliability (0; no agreement, slight; 0.2–0.4, Fair; 0.4–0.6, Good; 0.6–0.8, Excellent; 0.8 < 1, perfect; 1). Chi-square test was used to compare the distributions of frequencies across groups. A significant p-value was considered when it was equal to or less than 0.05.

## Results

### Patient demographics

A total of 236 patients with acetabular fractures were included in the study. The mean age of the cohort was 35.6 ± 13.9 years. Most patients were male (*n* = 197, 83.5%). Road traffic accident was the predominant mechanism of injury, accounting for 194 cases (82.2%), followed by falls from height in 26 patients (9.8%), falls on the ground in 11 patients (4.6%), and heavy object trauma in 5 patients (2.1%). Isolated acetabular fractures were observed in 145 patients (61.5%), while 78 patients (33%) sustained associated musculoskeletal injuries and 13 patients (5.5%) had associated non-musculoskeletal injuries.

### Characteristics and distribution of the modifiers (Tables 1, 2, 3)

Some modifiers were more common in older age groups, including head fracture (*p* = 0.041), head impaction (*p* = 0.042), roof impaction (*p* = 0.039), and Quadrilateral plate involvement (*p* = 0.044). Roof impaction was more common in T and unclassifiable fracture (*p* = 0.004). Marginal impaction was more common in posterior wall fracture (*p* = 0.012). Head impaction was most common in transverse and transverse with posterior wall (*p* = 0.022). Head fracture was more common with the posterior wall (*p* = 0.008). Intraarticular fragments were more common in posterior wall fracture (*p* < 0.001). Pelvic ring involvement was more common with anterior column, transverse and associated both-column fractures (*p* = 0.060). Quadrilateral plate involvement was more common in unclassifiable fractures, and both columns (*p* < 0.001), and fracture site comminution was more common in posterior wall fractures (*p* = 0.028).

We found posterior wall fractures associated with other fracture types, not included in the Letournel classification: both-column fracture in 1 case, T-fracture in 14 cases. Looking at individual fracture types, some are significantly associated with one or more modifiers. For example, a posterior wall fracture is commonly associated with posterior hip dislocation, fracture site comminution, intraarticular fragments, and marginal impaction (*p* < 0.001). Posterior column fracture is frequently associated with dislocation (*p* = 0.023). Anterior column fracture is commonly associated with pelvic ring involvement and roof impaction (*p* = 0.039). Transverse fracture is frequently associated with head and roof impaction, and pelvic ring involvement (*p* = 0.047). Transverse with posterior wall fracture is frequently related to intra-articular fragments, dislocation, fracture site comminution and head impaction (*p* < 0.001). Posterior column and posterior wall fracture is commonly associated with dislocation, Intra-articular fragment, and fracture site comminution. (*p* = 0.002). Both column fracture is commonly associated with fracture site comminution, quadrilateral plate, and pelvic ring involvement, and (*p* = 0.005). T fracture is commonly associated with hip dislocation, roof and head impaction (*p* = 0.012). Twenty-two fractures (9.3%) were considered unclassifiable by Letournel Classification^27^.Using specific modifiers enabled identification of the following unclassified fractures: the Quadrilateral plate modifier for the pure quadrilateral plate fracture (1) and Anterior column with quadrilateral plate (4), the posterior wall modifier for Both column with posterior wall (1) and T with posterior wall (14). This accounts for the identification of 20 out of 22 unclassified fractures (91%).

**Table 1:** Demonstratesthe incidence of each modifier.

Type of modifier	*n* = 236
Roof impaction	24 (10.2%)
Marginal impaction	18 (7.6%)
Head impaction	22 (9.3%)
Head fracture	11 (4.7%)
Intra-articular fragment	41 (17.4%)
Dislocation	96 (40.7%)
Central	25 (10.5%)
Anterior	2 (0.8%)
Posterior	69 (29.2%)
Fracture site comminution	51 (21.6%)
Pelvic ring involvement	20 (8.5%)
Quadrilateral plate involvement	18 (7.6%)
Posterior wall (in fractures other than posterior wall, post column with posterior wall, and transverse with posterior wall)	16 (6.8%)

**Table 2:** Demonstrates the relation of each modifier and fracture type.

Category	Roof impaction (*n* = 24)	Marginal impaction (*n* = 18)	Head impaction (*n* = 2 2)	Head fracture (*n* = 11)	Intra-articular fragments (*n* = 41)
Post. Wall	1 (4.2%)	**10 (55.6%)**	1 (4.5%)	**8 (72.7%)**	**18 (43.9%)**
Ant. Wall	0 (0%)	0 (0%)	0 (0%)	0 (0%)	1 (2.4%)
Post. Col.	2 (8.3%)	1 (5.6%)	0 (0%)	0 (0%)	0 (0%)
Ant. Col.	2 (8.3%)	0 (0%)	0 (0%)	0 (0%)	0 (0%)
Transverse	**4 (16.7%)**	0 (0%)	**5 (22.7%)**	0 (0%)	0 (0%)
Trans. & Post. Wall	1 (4.2%)	2 (11.1%)	**5 (22.7%)**	1 (9.1%)	9 (22%)
Post. Col. & Wall	1 (4.2%)	**3 (16.7%)**	**4 (18.2%)**	1 (9.1%)	**8 (19.5%)**
Both Columns	2 (8.3%)	0 (0%)	0 (0%)	0 (0%)	0 (0%)
T fracture	**5 (20.8%)**	0 (0%)	**4 (18.2%)**	0 (0%)	0 (0%)
Ant. & Post. Hemi.	1 (4.2%)	0 (0%)	0 (0%)	0 (0%)	0 (0%)
Unclassified	**5 (20.8%)**	2 (11.1%)	3 (13.6%)	1 (9.1%)	**5 (12.2%)**
P-value*	**= 0.004**	**= 0.012**	**= 0.022**	**= 0.008**	**< 0.001**

Category	Dislocation (*n* = 96)	Pelvic involvement (*n* = 20)	QLP involvement (*n* = 18)	Fracture site comminution (*n* = 51)	Posterior wall (*n* = 16)
Post. Wall	**38 (39.6%)**	0 (0%)	0 (0%)	**15 (22%)**	**-**
Ant. Wall	2 (2.1%)	1 (5%)	0 (0%)	2 (3.9%)	0 (0%)
Post. Col.	5 (5.2%)	0 (0%)	0 (0%)	0 (0%)	0 (0%)
Ant. Col.	1 (1%)	**4 (20%)**	0 (0%)	0 (0%)	0 (0%)
Transverse	**6 (6.3%)**	**4 (20%)**	0 (0%)	0 (0%)	0 (0%)
Trans. & Post. Wall	**8 (8.3%)**	3 (15%)	0 (0%)	**7 (13.7%)**	-
Post. Col. & Wall	**12 (12.5%)**	0 (0%)	1 (5.6%)	**5 (9.8%)**	-
Both columns	4 (4.2%)	**4 (20%)**	**5 (27.8%)**	**11 (21.6%)**	0 (0%)
T fracture	8 (8.3%)	3 (15%)	**3 (16.7%)**	1 (2%)	0 (0%)
Ant. & Post. Hemi.	0 (0%)	0 (0%)	1 (5.6%)	0 (0%)	0 (0%)
Unclassified	**12 (12.5%)**	1 (5%)	**8 (44.4%)**	**010 (19.6%)**	**16 (100%)**
P-value*	**= 0.010**	= 0.060	**< 0.001**	**= 0.028**	**= 0.009**

*Chi-square analysis was used to compare the frequency among groups.

**Table 3:** Demonstrates the relation of each modifier and fracture type.

Category	Post. Wall (*n* = 52)	Ant. Wall (*n* = 3)	Post. Col. (*n* = 15)	Ant. Col. (*n* = 22)	Transverse (*n* = 34)	Trans. + Post. Wall (*n* = 20)
Head fracture	8 (15.4%)	0 (0%)	0 (0%)	0 (0%)	0 (0%)	1 (5%)
Marginal impaction	10 (19.2%)	0 (0%)	1 (6.7%)	0 (0%)	0 (0%)	2 (10%)
Quadrilateral plate Involvement	0 (0%)	0 (0%)	0 (0%)	0 (0%)	0 (0%)	0 (0%)
Pelvic ring involvement	0 (0%)	**1 (33.3%)**	0 (0%)	**4 (18.2%)**	**4 (11.8%)**	3 (15%)
Head impaction	1 (1.9%)	0 (0%)	0 (0%)	0 (0%)	**5 (14.7%)**	5 (25%)
Roof impaction	1 (1.9%)	0 (0%)	**2 (13.3%)**	**2 (13.3%)**	**4 (11.8%)**	1 (5%)
Intra-articular fragment	**18 (34.8%)**	**1 (33.3%)**	0 (0%)	0 (0%)	0 (0%)	**9 (45%)**
Fracture site comminution	**15 (28.8%)**	**2 (66.7%)**	0 (0%)	0 (0%)	0 (0%)	**7 (35%)**
Posterior wall	**-**	0 (0%)	0 (0%)	0 (0%)	0(0%)	**-**
Dislocation	**38 (73.1%)**	**2 (66.7%)**	**5 (33.3%)**	1 (4.5%)	**6 (17.6%)**	**8 (40%)**
P-value*	**< 0.001**	**= 0.001**	**= 0.023**	**= 0.039**	**= 0.047**	**< 0.001**

Category	Post. Col. & Wall (*n* = 15)	Both Col. (*n* = 28)	T Fracture (*n* = 19)	Ant. & Post. Hemi-transverse (*n* = 5)	Unclassified (*n* = 22)	
Head fracture	1 (6.7%)	0 (0%)	0 (0%)	0 (0%)	1 (4.3%)	
Marginal impaction	3 (20%)	0 (0%)	0 (0%)	0 (0%)	2 (8.7%)	
Quadrilat. plate Involvement	1 (6.7%)	**5 (17.9%)**	3 (15.8%)	**1 (20%)**	**8 (34.8%)**	
Pelvic ring involvement	0 (0%)	4 (14.3%)	3 (15.8%)	0 (0%)	1 (4.3%)	
Head impaction	4 (26.7%)	0 (0%)	**4 (21.1%)**	0 (0%)	3 (13%)	
Roof impaction	1 (6.7%)	2 (7.1%)	**5 (26.3%)**	**1 (20%)**	5 (21.7%)	
Intra-articular fragment	**8 (53.3%)**	0 (0%)	0 (0%)	0 (0%)	5 (21.7%)	
Fracture site comminution	**5 (33.3%)**	**11 (39.3%)**	1 (5.3%)	0 (0%)	**10 (43.5%)**	
Posterior wall	**-**	0 (0%)	0 (0%)	0 (0%)	**16 (73.9%)**	
Dislocation	**12 (80%)**	4 (14.3%)	**8 (42.1%)**	0 (0%)	**12 (52.2%)**	
P-value*	**= 0.002**	**= 0.005**	**= 0.012**	**= 0.044**	**< 0.001**	

*Chi-square analysis was used to compare the frequency among groups.

### Influence on surgical decision making

We used surgical hip dislocation in 21 cases (8.8%) based on certain modifiers, namely,

femoral head fracture (*n* = 11), roof impaction (*n* = 6), and intraarticular fragment (*n* = 2), pure impaction (*n* = 1), and labral avulsion with posterior rim (*n* = 1). Infrapectineal plating was done in 14 cases (6%) based on the presence of the quadrilateral plate modifier. All 20 cases (7.6%) with pelvic ring involvement needed an additional surgical plan/or surgical approach for additional fixation to the pelvic ring. An additional posterior approach was added to the modified Stoppa for the case of both-column fracture with a posterior wall (one of the unclassified fractures). For the 14 cases of T with posterior wall involvement, the presence of posterior wall involvement mandated the use of the posterior approach alone in 12 cases; however, in 2 cases, the posterior approach was combined with the modified Stoppa approach. We used a supraacetabular bone window to reduce the roof impaction in 3 cases (2 both columns and one anterior with posterior hemitransverse.

### Interrater reliability (Table 4)

The interrater reliability ranged from good to perfect agreement for all modifiers, with a weighted Kappa coefficient ranging from 0.727 to 1, *p* < 0.001, except fracture-site comminution, which showed fair agreement with a weighted Kappa coefficient of 0.519, *p* < 0.001.

**Table 4:** Interobserver reliability.

Modifier	weighted kappa	*P* value
Roof impaction	**0.742**	*P* < 0.001
Marginal impaction	**0.896**	*P* < 0.001
Head impaction	**0.727**	*P* < 0.001
Head fracture	**0.938**	*P* < 0.001
Intraarticular fragment	**0.895**	*P* < 0.001
Dislocation	**1.000**	*P* < 0.001
Fracture site comminution	**0.519**	*P* < 0.001
Pelvic ring involvement	**0.911**	*P* < 0.001
Quadrilateral plate involvement	**0.892**	*P* < 0.001
Posterior wall	**0.870**	*P* < 0.001

## Discussion

The ideal classification system should include the majority of fracture types, be easy to master, and have satisfactory inter-observer and intra-observer reliability^28–30^. Moreover, it should also give adequate guidance for the surgical procedure.

Since its introduction in 1960, Letournel classification has been the most widely used classification for acetabular fractures. Although many other classification systems^3,21,31^ have been proposed to overcome the shortcomings of Letournel classification in terms of difficulty, limited prognostic prediction and high incidence of unclassifiable fractures, Letournel classification is still the gold standard for acetabular classification. Efforts should be directed towards teaching and simplifying the Letournel classification using advanced imaging, 3D modelling, and algorithms^9,32–40^ rather than towards establishing a new classification system. A new or unclassified fracture may require subclassification within the Letournel classification^18,20,41,42^.

In Concordance with many authors, we considered the Letournel classification to be the cornerstone of acetabular fracture classification. However, we suggest adding modifiers to address its shortcomings, namely, limited predictive power and reducing the number of unclassified variants. Additionally, some modifiers favour the use of special techniques, such as infrapectineal plating or approaches like surgical hip dislocation.

### Prognostic implications

Acetabular cartilage impaction has been shown to negatively affect the outcome after fixation of acetabular fractures^43–46^. Anglen et al.^46^ described the gull-wing sign, a superomedial roof impaction of osteopenic bone in the elderly. The presence of the Gull wing sign carries the risk of inadequate reduction and a subsequent poor prognosis^46,47^. However, impaction injuries can occur anywhere in the acetabulum, not only the dome^48,49^. Marginal impaction has also been identified as an independent risk factor for bad outcomes^45,48,50^. Moreover, fracture comminution (more than three fragments), intra-articular fragments, femoral head injuries, and hip dislocation can adversely affect the outcome even after anatomical reduction. It should be carefully addressed in the surgical plan^22–24,43,49–56^. Further studies are needed to confirm the individual long-term effects of these modifiers on the outcome of acetabular fractures.

### Inclusivity

Unclassifiable fractures using the Letournel classification were reported in many studies and ranged from 1%^18,57^ to 35%^20^. In their series of 100 acetabular fractures, Hutt et al.^20^ found 35 acetabular fractures (35%) to be unclassifiable by the Letournel system. The majority of these fractures were common in older age groups with osteopenic bone. Fractures involving the anterior column together with the quadrilateral surface were the most common form of unclassifiable fractures. In this series, fractures of the anterior column with the quadrilateral plate comprised 4 (18.2%) of the unclassified fractures.

Fracture of the acetabulum in the elderly usually results from low-energy trauma. Usually, it shows a different pattern of fractures not included in Letournel classification involving mainly the quadrilateral plate, either alone or in combination with the anterior column^20^. The addition of the quadrilateral plate as a separate modifier to the Letournel classification addresses this problem. We used the definition advocated by Elnahal et al.^26^ to define the quadrilateral plate fracture as a complete or partial separation of the quadrilateral plate from either the anterior or posterior column. By definition, quadrilateral plate involvement may necessitate the addition of the infrapectineal plate to reduce the quadrilateral plate. This is another advantage of adding the quadrilateral plate as a modifier.

Posterior wall fractures may accompany elementary fracture types in the Letournel classification, such as posterior column and transverse fractures, and are considered associated types. However, a posterior wall fracture may accompany other fracture types, such as column fractures and T fractures^31^. Although these combinations are not uncommon, they are not included in the Letournel classification. Adding a posterior wall fracture modifier to the Letournel classification may solve this problem. In this series, the posterior wall was associated with both column fracture in 1 (4.5%) and with T fracture in 14 (63.6%) of the unclassified fractures.

So, using the quadrilateral plate and posterior wall as modifiers addresses the Letournel classification’s limited coverage. In this series^27^, the number of unclassified fractures was reduced by 90%.

### Guide for the optimal surgical approach

The presence of articular impaction represents not only a bad prognostic sign but also a surgical difficulty. Access to the impacted articular surface may be difficult because of the incomplete fracture line or because of the surgical approach itself, which does not provide full access to the area of impaction^58^.

Specific surgical approaches and techniques have been described to improve visualisation and/or reduce the impact of impacted articular surfaces, such as surgical hip dislocation^59^, the extended iliofemoral approach^60^, and the iliac cortical window^58^.

Surgical hip dislocation gives a 360-degree access to the acetabulum and head of the femur, which enables easy access to the impacted articular surface. Moreover, it is helpful in other conditions, such as fracture head of femur, entrapped posterior wall, incarcerated intraarticular fragments, reduction and fixation of the anterior column, and labral injuries^59,61–63^.

In our cohort, specific modifiers, such as femoral head fracture, roof impaction, and intraarticular fragment, led us to select surgical hip dislocation as the appropriate approach to manage the acetabular fracture in 21 (8.8%) cases.

Pelvic ring involvement may alter the surgical approach accordingly and has its own impact on the overall outcome of the acetabular fracture. Similarly, the association of the posterior wall may alter the surgical approach in T and both-column fractures.

The presence of a Quadrilateral plate fracture requires adequate rigid fixation and buttressing to prevent medial dislocation of the femoral head. This might be difficult to achieve through the classic ilioinguinal approach. Some surgeons prefer the modified Stoppa approach, which provides direct access for reduction and fixation of the fractured quadrilateral plate. Infrapectineal plating is a suitable method for buttressing a broken quadrilateral plate with a low risk of late loss of reduction^47,64^.

This study showed an association between specific modifiers and certain fracture types. For example, Roof impaction was more common in the T type. This might be due to the undisplaced roof in T fracture, which remains attached to the axial skeleton, unlike both column fractures, so the head strikes against the roof, causing impaction. This could explain the poor prognosis of T fractures. Similarly, head impaction is common in transverse fractures and in transverse fractures with posterior wall fractures. Fracture head, intra-articular fragment, and fracture site comminution are more common with posterior wall fractures. Therefore, these modifiers should be sought in every case of posterior wall fracture.

The main limitation of this study is the lack of follow-up, which limited our ability study the relation between each modifier and the final prognosis of each fracture pattern. However, this study can serve as a basis for a multicenter, long-term survival study to assess the effect and relative weight of each modifier on the prognosis of acetabular fractures.

The identity of the acetabular fracture is more critical than any classification system. Identifying the fracture means providing an accurate description of the acetabular fracture, including possible associated types and morphological patterns (modifiers such as posterior wall, quadrilateral plate, articular impaction, etc.). This helps plan the best surgical approach and assess the possible prognosis in light of the present modifiers. Gathering all these modifiers with Letournel classification categories to form ‘the Acetabular Fracture Identification Sheet “AFIS” will be more helpful than adopting a new or modified classification system. Alternatively, the new modifier section can be added as an addendum to the original Letournal classification.

## Conclusion

Accurate identification of fracture characteristics significantly enhances the value of acetabular fracture classification. This process involves providing a detailed description of the fracture, including all possible combinations, such as posterior wall involvement and quadrilateral plate displacement. Such precision aids in selecting the most appropriate surgical approach and assessing the prognosis based on the identified modifiers. Incorporating modifiers enhances the Letournel classification by increasing its comprehensiveness, ultimately providing better guidance for surgical planning.

## Data Availability

Data are available from the authors upon reasonable request.
